# Recrudescence of a FLT3 wild-type CMML clone after allogeneic stem cell transplant for FLT3-ITD acute myeloid leukemia

**DOI:** 10.1093/oncolo/oyag213

**Published:** 2026-06-02

**Authors:** Hatem A Ellaithy, Valentina Nardi, Christopher B Hergott, Yi-Bin Chen

**Affiliations:** Hematopoietic Cell Transplant & Cell Therapy Program, Mass General Brigham Cancer Institute, Boston, MA, 02114, United States; Department of Pathology, Massachusetts General Hospital, Boston, MA, Boston, MA, 02114, United States; Department of Pathology, Brigham and Women’s Hospital, Boston, MA, 02115, United States; Hematopoietic Cell Transplant & Cell Therapy Program, Mass General Brigham Cancer Institute, Boston, MA, 02114, United States

**Keywords:** acute myeloid leukemia, chronic myelomonocytic leukemia, clonal evolution, allogeneic hematopoietic cell transplantation, FLT3-ITD

## Abstract

Late relapse after allogeneic hematopoietic cell transplantation (HCT) with phenotypic and molecular divergence from the original leukemia is rare. We describe a 60-year-old man with *FLT3-*ITD–mutated acute myeloid leukemia (AML) who achieved durable remission following venetoclax-based therapy and a combined HLA-matched sibling HCT–kidney transplant with *FLT3* inhibitor maintenance. Four years post-transplant, he developed chronic myelomonocytic leukemia (CMML-1) characterized by re-emergence of driver mutations without *FLT3-*ITD, marked loss of donor myeloid chimerism, preserved donor T-cell chimerism, and sustained renal allograft function. This case highlights a unique clinical circumstance that may function to recontextualize myelomonocytic features in AML: that they can be attributed to acute leukemias arising from clonal hematopoiesis or occult chronic malignancies, as opposed to de novo AML, particularly given the difficulty in differentiating the two in the acute leukemic setting.

Key points

- Phenotypic divergence at post-HCT relapse can unmask the true underlying disease.
- Myelomonocytic features in apparent de novo AML may reflect transformation from occult CMML or CMML-like clonal hematopoiesis, which carries different relapse biology than de novo AML.
- Lineage-specific chimerism shapes outcomes in combined HCT–solid organ transplantation.

## Patient story

**Figure 1.**
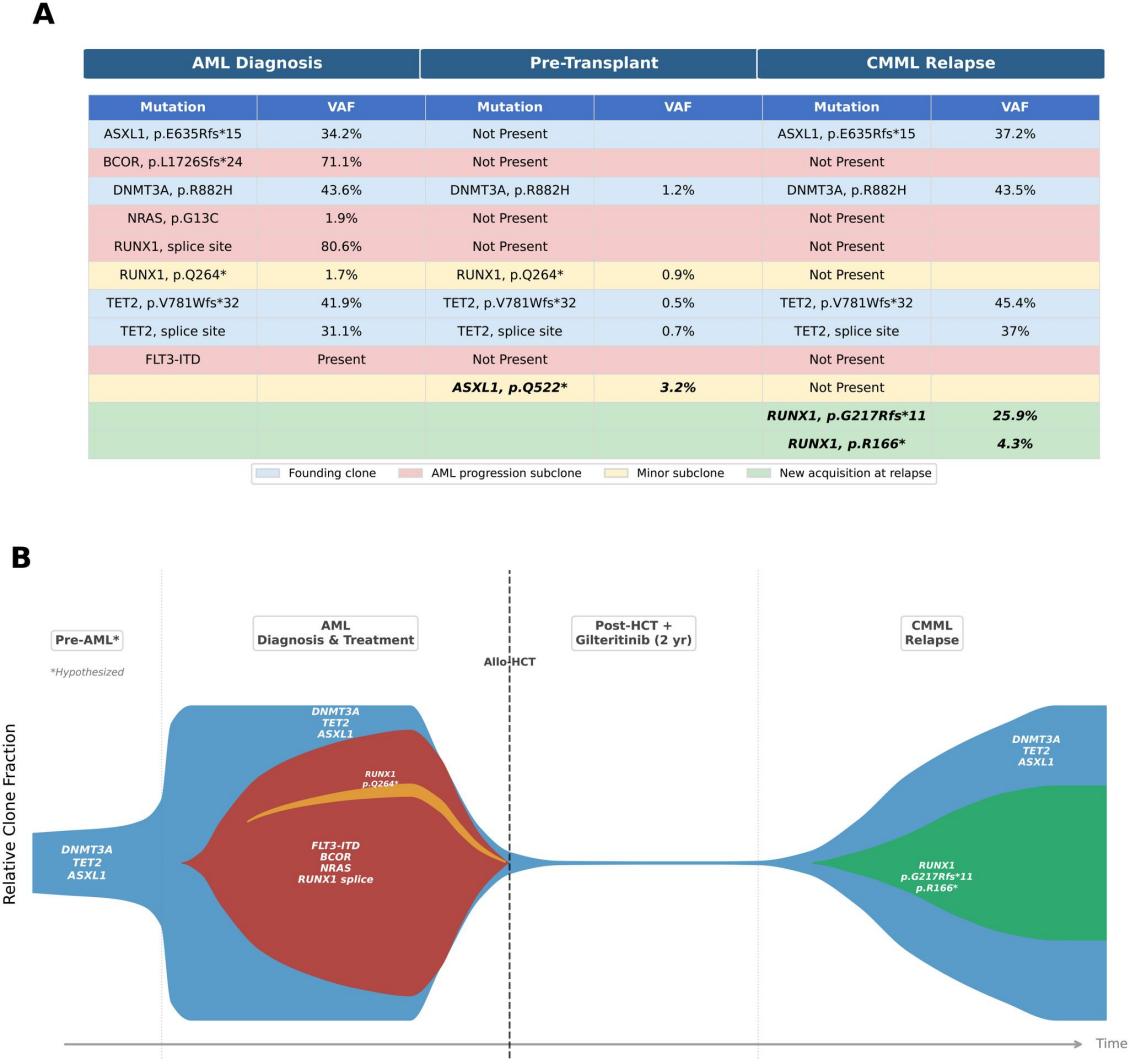
(A) Rapid Heme Panel at diagnosis, post-induction chemotherapy/pre-transplant and at relapse. Corresponding fish plot highlighting our proposed clonal evolution and founding clone. (B) Fish plot illustrating the hypothesized clonal dynamics of the founding epigenetic clone (*DNMT3A*, *TET2*, *ASXL1*) across four disease phases. In the pre-AML phase (hypothesized), the founding clone exists as a subclinical entity with CMML-like potential. At AML diagnosis, a *FLT3-*ITD–driven progression subclone rapidly emerges and dominates the disease, harboring co-occurring *BCOR*, *NRAS*, and *RUNX1* splice site mutations, with a minor *RUNX1* p.Q264* subclone. Following allogeneic HCT and two years of gilteritinib maintenance, the progression subclone is eradicated while the founding clone persists at a minimal residual level. At CMML relapse (∼4 years post-HCT), the founding clone re-emerges at near-diagnostic variant allele frequencies, now with acquisition of two novel *RUNX1* mutations (p.G217Rfs*11 & p.R166*) representing de novo clonal evolution along a myelomonocytic differentiation trajectory distinct from the original AML transformation. Clone sizes are schematic and not drawn to scale. *Created with the assistance of Claude (Anthropic)*.

A 60-year-old male with a history of mild hypertension and hyperlipidemia presented to the emergency room with several weeks of dyspnea on exertion and lightheadedness. The patient was found to have leukocytosis to 65,000 cells/µL with 35% blasts and 45% monocytes on peripheral blood differential count. He was transferred to a tertiary center due to concern for acute myeloid leukemia (AML) and initiated on hydroxyurea for cytoreduction. A diagnostic bone marrow biopsy was performed, which showed 83% blasts with myelomonocytic features, a normal karyotype, and mutations in *ASXL1*, *BCOR*, *DNMT3A*, *NRAS*, *RUNX1*, *TET2*, and *FLT3* internal tandem duplication (*FLT3*-ITD) (Figure 1A). He subsequently developed tumor lysis syndrome and an inflammatory surge leading to severe shock, acute hypoxemic respiratory failure and renal failure, requiring transfer to the medical ICU for vasopressor support, intubation and mechanical ventilation and continuous renal replacement therapy. He was treated with broad-spectrum antimicrobials and high-dose corticosteroids, with rapid reversal of his respiratory failure but persistence of his renal failure. The etiology of his multi-organ decompensation was thought to be related to his high burden of disease and tumor lysis upon cytoreduction.

Induction chemotherapy was initiated with bolus daunorubicin and continuous infusion cytarabine (7 + 3) followed by the oral FLT3 inhibitor midostaurin. He responded well to therapy initially with clearance of circulating blasts and improvement of his white blood cell count, but experienced frank disease progression with reemergence of peripheral blasts 3 weeks after initiation of therapy. Upon disease relapse, he developed a cytokine release-like syndrome with severe hypotension and subsequent respiratory failure requiring ICU transfer for vasopressor support and mechanical ventilation. He was initiated on venetoclax and decitabine salvage chemotherapy and treated with tocilizumab and high-dose corticosteroids with rapid improvement of his hemodynamic collapse and respiratory failure. His hospitalization was further complicated by myopericarditis, atrial fibrillation, shock liver, pulmonary embolism, heart block, and a gastrointestinal bleed. After medical and hematopoietic recovery, a bone marrow biopsy showed complete remission (CR) without detectable *FLT3*-ITD-based minimal residual disease (MRD).

He was discharged from the hospital after a 64-day inpatient admission still requiring hemodialysis, as his renal function did not recover. He was then referred to our hospital for consideration for combined hematopoietic cell–kidney transplantation from the same donor.^1^ His pre-transplant workup identified a Gleason 3 + 4 prostate cancer which was treated with prostatectomy. He continued decitabine and venetoclax as a bridge to transplant. His sister was found to be fully HLA-matched and ABO-compatible, and he underwent a combined bone marrow-kidney transplant ∼15 months after his initial leukemic presentation. He received reduced-intensity conditioning with cyclophosphamide (14.5 mg/kg) days −6, −5, fludarabine (24 mg/m2) IV daily, days −4, −3, −2, and total body irradiation 200 cGy twice on day −1. Extended dialysis was given after each fludarabine dose as we have published.^2^ For graft-versus-host disease (GVHD) prophylaxis, he received high-dose cyclophosphamide (50 mg/kg) IV daily on days +3 and +4 with mesna and intravenous fluid support. He was started on mycophenolate mofetil and tacrolimus on day +5. His post-transplant course was relatively uncomplicated. Maintenance gilteritinib was initiated after engraftment and continued for 2 years post-transplant. His day 100 and 1-year bone marrow biopsies showed complete remission with 100% donor chimerism in the bone marrow and peripheral blood and without detectable disease by *FLT3*-ITD-based MRD assays and no residual mutations on conventional next-generation sequencing (NGS). No further molecular testing, including NGS, chimerism, or *FLT3-*ITD MRD was checked again after year 1 in accordance with our standard of care at that time and post-transplant monitoring guidelines.^3^ His kidney allograft functioned well and he had been tapered off all immunosuppression by 6 months post-transplant without any emergent acute or chronic GVHD.

**Figure 2.**
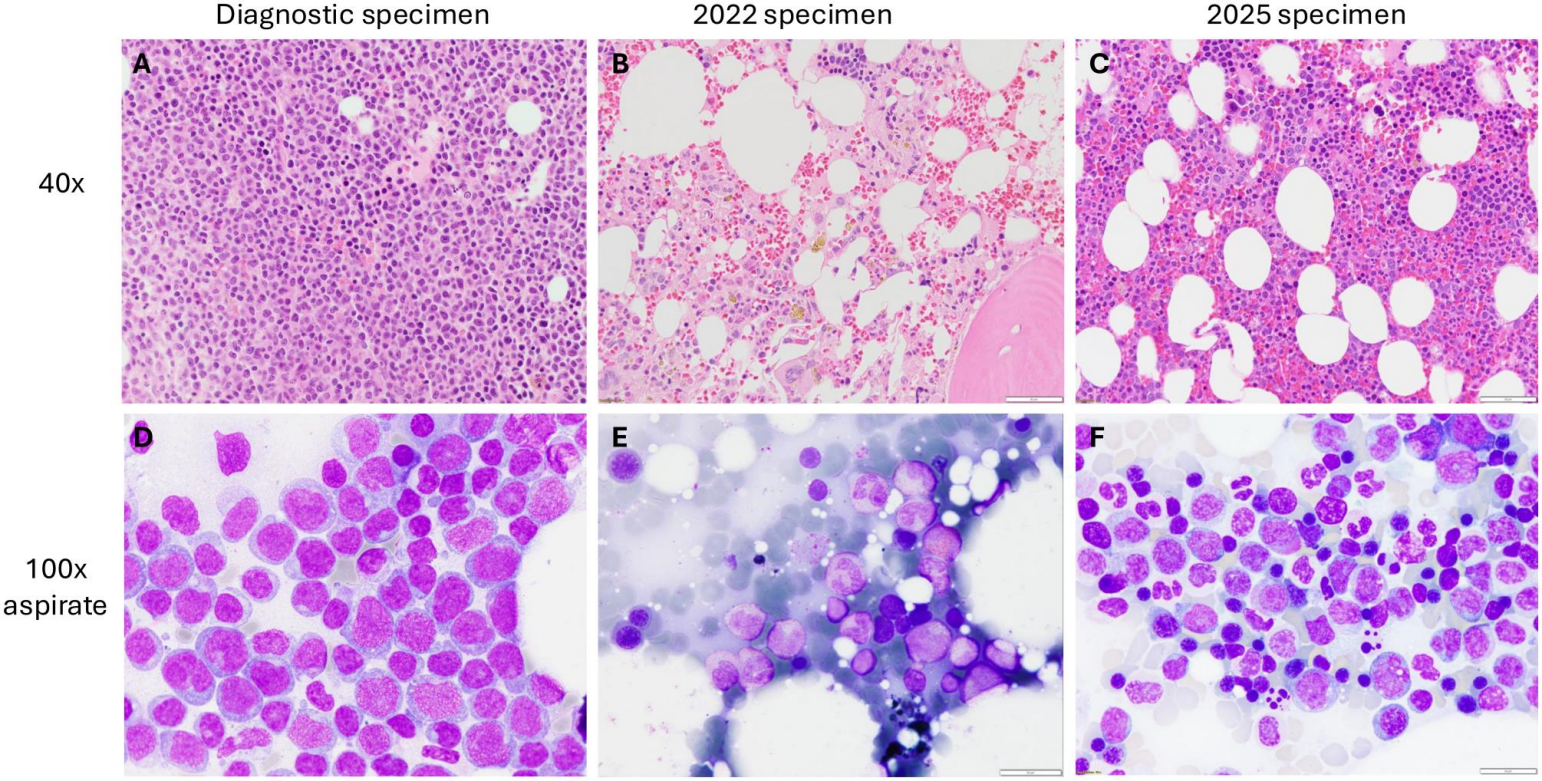
Serial bone marrow core/clot specimens (A-C; H&E, 40×) and bone marrow aspirate smears (D-F; 100×). Initial diagnostic samples (A & D) demonstrate hypercellular sheets of monotonous blasts with monocytic features, consistent with acute myeloid leukemia (AML). Post-treatment interim samples (B & E) show reduction of marrow cellularity and maturing hematopoiesis. Relapse specimen (C & F) demonstrates a hypercellular proliferation of dysplastic, maturing myelomonocytic forms and 3% blasts, consistent with chronic myelomonocytic leukemia-1 (CMML-1).

He continued to do well until ∼4 years after combined transplant, when he developed mild thrombocytopenia which persisted over several months. He underwent diagnostic bone marrow biopsy that showed a hypercellular, myeloid-dominant marrow with increased and abnormal maturing myelomonocytic forms with 3% blasts, consistent with chronic myelomonocytic leukemia (CMML-1) (Figure 2). Mutational analysis showed recurrence of *ASXL1*, *DNMT3A*, and *TET2* mutations and new *RUNX1* mutations (Figure 1A). No *FLT3*-ITD mutation was identified. Bone marrow analysis now showed 5% donor chimerism and 25% donor chimerism in unseparated peripheral blood but fractionated peripheral blood showed 98% CD3+ donor chimerism. *FLT3*-ITD MRD testing was performed and showed no evidence of *FLT3*-ITD to 10^−5^. His kidney graft continued to function well with no evidence of rejection.

## Molecular tumor board

### Genotyping results and interpretation of the molecular results

This patient’s current presentation is consistent with late relapse of a myeloid malignancy after allogeneic hematopoietic cell transplantation. Interestingly, his relapse is most morphologically and molecularly consistent with CMML-1 as opposed to his initial presenting diagnosis of AML. The central question here is whether this patient had undiagnosed CMML initially and presented to care at the time of leukemic transformation or whether he had de novo AML with myelomonocytic differentiation. Blood counts prior to his original diagnosis are sparse, but complete blood counts performed one and three years prior to AML diagnosis showed a normal white blood cell count. No differential was obtained to evaluate his peripheral monocyte count. His original diagnostic bone marrow biopsy exhibited myelomonocytic differentiation which is often seen with CMML transformed to AML, but can also be seen in de novo AMLs. His original mutational profile, aside from the *FLT3*-ITD, was consistent with common molecular abnormalities seen in cases of CMML.^4^  *FLT3*-ITD mutations are present in approximately 25%-30% of AML cases^5^ and are thought to be late mutation acquisitions that function as a driver for progression to leukemia.^6^

While it is difficult to reverse engineer his molecular picture based on the available data, we hypothesize that he had underlying, subclinical CMML (or pre-leukemic clone with CMML-like potential) that transformed to a secondary AML with the acquisition of a *FLT3*-ITD driver mutation (Figure 1B). Clonal evolution that leads to leukemic transformation often begins with driver mutations (often 1-6) in a founding clone and eventual acquisition of a progression mutation (such as *FLT3*-ITD) that drives rapid expansion and maturation arrest.^7^^,^^8^ The two years post-transplant that he was treated with a FLT3 inhibitor may have served to diminish his risk for relapsing with FLT3-ITD AML,^9^ but offered little protection against CMML leading to recrudescence of this founding clone as evidenced by re-emergence of his original ASXL1, DNMT3A and TET2 mutations at near identical variant allele fractions (VAF). Furthermore, the acquisition of other clones (eg, RUNX1) suggests clonal evolution of the antecedent CMML.

### Functional and clinical significance of the specific mutation

CMML is often underdiagnosed, particularly the dysplastic types, if monocytosis is subtle.^10^ Mutational analysis and flow cytometric analysis that evaluate different monocyte populations (increase in the MO1 fraction (CD14+ CD16−) above 94% and a decrease in the percentage of MO3 (CD14− CD16+) to <1.13%) can aid in diagnosis, but often require expert pathologist interpretation at high-volume centers (see Figure 1).^11^ In any patient presenting with CMML, the mutational profile and laboratory abnormalities determine the selection of treatment. Our patient had only mild thrombocytopenia and was otherwise asymptomatic, but harbored high-risk mutations, giving him a CMML CPSS-Mol (risk score) of 4 (ASXL1, RUNX1, transfusional need) which corresponds with a 48% cumulative incidence of AML at 48 months and a median overall survival of 18 months.^12^ Moreover, and more importantly, relapsed disease after HCT and a likely previous transforming event (*FLT3*-ITD acquisition) confer a very high risk of recurrent progression. Further complicating this case is the presence of his renal allograft, which does not require long-term immunosuppression due to its genotypical concordance with his donor-derived immune system. T-cell chimerism often does drop once patients relapse,^13^ and can be an early predictor for those who experience relapse after transplant.^14^ Thankfully for our patient, his peripheral T-cell chimerism remained at nearly 100% donor origin even at disease relapse, which engendered natural tolerance and protected his kidney from allograft rejection.

Cumulative incidence of relapse after HCT for CMML is cited to be as high as 50%,^15^ with an overall mortality that ranges widely depending on the biological risk profile of the disease. It is difficult to quantify his CMML relapse risk given his original presentation of frank AML. Moreover, the temporality of clonal evolution is not clear given his largely normal blood counts, even after relapse. His mutational profile is mostly in genes responsible for histone modifications (*ASXL1*), DNA methylation (*DNMT3A* and *TET2*), signaling pathway mutations (*NRAS*), and transcription factor mutations (*RUNX1*). Of his mutations in epigenetic modifiers (*ASXL1*, *DNMT3A* and *TET2*), the former two may confer higher-risk features, whereas *TET2* mutations may associate with better prognosis.^4^^,^^16^ Interestingly, *ASXL1* and *DNMT3A* mutations have been associated with anemia, leukocytosis, complex karyotypes, and extramedullary disease, none of which were observed in our patient.^4^^,^^17^  *TET2* is the most common mutation seen in CMML and is often associated with the dysplastic subtype.^12^ Mutations in *TET2* seem to cause a differentiation bias toward granulomonocytic expansion and away from erythroid differentiation.^16^  *RAS* mutations of any type are present in nearly 30% of CMMLs and often drive a proliferative CMML phenotype.^18^  *RUNX1* mutations are associated with thrombocytopenia, which was seen in our patient, and imply worse prognosis.^19^ While *FLT3* mutations also affect signaling pathways, they are quite uncommon in CMML and more frequently herald progression to AML.^20^

### Potential strategies to target the pathway and implications for clinical practice

Standard of care for relapse after HCT includes tapering of any present immunosuppression, chemotherapy and targeted agents to achieve remission followed by consolidation with some cellular therapy—either donor lymphocyte infusion (DLI) or a second transplant, usually from a different donor.^21^ A complicating factor for our patient is his kidney transplant (derived from his original donor). This would make a consolidative second transplant from a different donor less attractive, as it would require ongoing immunosuppression to prevent renal allograft rejection.

Historically, a subset of relapses after HCT possessing *FLT3*-ITD were able to achieve durable remissions with FLT3 inhibitor with or without DLI.^22^^,^^23^ Unfortunately, our patient’s current molecular profile did not reveal any targetable mutations. Standard chemotherapy using hypomethylating agents (HMA) or venetoclax are also options for post-transplant relapse.^24^

## Patient update

The patient continued to feel well with minimal symptoms from his relapse. He was initiated on decitabine for treatment of CMML.^25^ A tentative plan, if he achieved a disease response and return of donor-derived hematopoiesis, was to continue with several cycles of decitabine and consolidate with serial escalating-dose therapeutic DLIs in efforts to achieve durable remission and maintain renal allograft tolerance. However, his chimerism did not revert with decitabine therapy and we proceeded to second allogeneic transplant using the same donor—this one with reduced intensity conditioning comprised of fludarabine/melphalan followed by matched related donor peripheral blood progenitor cells and GVHD prophylaxis with high-dose cyclophosphamide paired with sirolimus/mycophenolate mofetil. He is now 2 months after second HCT with full donor hematopoietic chimerism and intact renal function.

## Data Availability

The data used in this manuscript is not shared publicly due to patient privacy concerns. Some of the data can be shared by corresponding authors upon request.
